# Quality by Design‐Based Development of a Robust LC Method for Simultaneous Estimation of Process‐ and Degradation‐Related Impurities in Rifapentine Drug Product for the Treatment of Active and Latent Tuberculosis

**DOI:** 10.1002/bmc.70430

**Published:** 2026-03-22

**Authors:** Siva Prasad Korikana, Sreenivasa Rao Battula, Naresh Konduru, Aravind Kurnool, Divya Kumar Vemuri, Venkata Lakshamana Sagar Dantinapalli

**Affiliations:** ^1^ Department of Chemistry GITAM School of Science, GITAM (Deemed to be University) Visakhapatnam Andhra Pradesh India; ^2^ Department of Chemistry, Sridevi Women's Engineering College Science and Humanities Hyderabad Telangana India; ^3^ Department of Chemistry, GITAM School of Science GITAM Deemed to be University Hyderabad Telangana India; ^4^ Department of Chemistry Raffles University Alwar Rajasthan India

**Keywords:** analytical QbD principles, and forced degradation studies, high‐performance liquid chromatography technique, rifapentine‐related substances, treatment of latent tuberculosis

## Abstract

A precise, robust, and stability‐indicating liquid chromatographic (LC) method coupled with a photodiode array (PDA) detector was developed and validated for the quantitative estimation of rifapentine, an essential therapeutic agent for both active and latent tuberculosis (TB). The chromatographic separation was achieved on a YMC Pack C18 column (250 × 4.6 mm, 5 μm) using a gradient elution with Solvent A composed of phosphate buffer (pH 6.3) and acetonitrile (90:10, v/v), and Solvent B consisting of 100% acetonitrile. The optimized parameters included a flow rate of 1.0 mL min^−1^, a column temperature of 30 °C, an injection volume of 25 μL, and UV detection at 330 nm. Method validation, conducted as per ICH Q2(R2) guidelines, confirmed excellent accuracy with recovery results between 97.0% and 103.0%. The method demonstrated linearity over a broad concentration range, from the limit of quantification (LOQ) to 200% of the target concentration, with correlation coefficients (*R* ^2^) exceeding 0.998 for all impurities. Precision, expressed as relative standard deviation (RSD), was consistently below 5.0%. Forced degradation studies indicated that rifapentine undergoes degradation under acidic, basic, oxidative, thermal, and photolytic conditions, thus confirming the stability‐indicating nature of the developed LC method.

## 1 Introduction

Rifapentine is a rifamycin‐class antimycobacterial medication used in combination with other antituberculosis drugs for the treatment of pulmonary tuberculosis caused by *Mycobacterium tuberculosis* . It is specifically recommended for use in patients with drug‐susceptible TB as part of a multi‐drug treatment regimen consisting of an initial 2‐month intensive phase followed by a 4‐month continuation phase. Rifapentine must not be used as monotherapy during either phase of treatment.

During the initial phase (2 months), the recommended dose is 600 mg twice weekly, administered under direct observation, with a minimum interval of 72 h between doses, in combination with appropriate antituberculosis agents. In the continuation phase (4 months), the recommended dose is 600 mg once weekly, also under direct observation, given along with isoniazid or another suitable companion antituberculosis drug. This structured dosing regimen ensures effective bacterial eradication and helps prevent the development of drug resistance.

The chemical name of rifapentine is rifamycin, 3‐[N‐(4‐cyclopentyl‐1‐piperazinyl)formimidoyl]rifamycin, also known as 5,6,9,17,19,21‐hexahydroxy‐23‐methoxy‐2,4,12,16,18,20,22‐heptamethyl‐8‐[N‐(4‐cyclopentyl‐1‐piperazinyl)‐formimidoyl]‐2,7‐(epoxypentadeca[1,11,13]trienimino)naphtho[2,1‐b]furan‐1,11(2H)‐dione 21‐acetate.

Rifapentine, a derivative of the rifamycin class of antibiotics, exhibits antimicrobial activity comparable to rifampin (rifampicin). The compound possesses a molecular formula of C₄₇H₆₄N₄O₁₂ and a molecular weight of 877.04 g/mol. Throughout the formulation development and optimization stages of the rifapentine drug product, no unknown or unspecified degradation products were detected. Forced degradation studies were further performed on the tablet dosage form, during which both process‐related and degradation‐related impurities emerged under a range of chemical and physical stress conditions. These impurities, referred to as Impurity 1 through Impurity 4, were confirmed to be structurally related to rifapentine and are therefore considered key indicators for impurity profiling and quality assessment of the finished product.

A detailed review of the published literature revealed multiple analytical approaches, such as HPLC, LC–MS, and LC–MS/MS, that have been previously applied for rifapentine evaluation [Ansari et al. 2013; Ansari et al. 2014; Ghantiwala et al. 2024; Goutal et al. 2016; Madhusudhan Reddy and Prasanna 2017; Mkhize et al. 2022; Qiyao et al. 2024; Winchester et al. 2015]. However, existing methods lack the required selectivity and sensitivity to quantify the rifapentine‐related impurities listed in Table 1, and no pharmacopeial procedure is currently available for this purpose. Consequently, there is a strong need to establish a novel, reliable, and stability‐indicating analytical method to accurately quantify rifapentine impurities and evaluate the drug's degradation behavior under various stress conditions.

**TABLE 1:** Comparison of existing methods and the proposed method.

S.No	Title of the existing research articles	Observations and disadvantages	Name of the journal and published year
1	Development and validation of UV/Visible spectrophotometric method for the estimation of rifapentine in bulk and pharmaceutical formulations.	Did not discuss forced degradation studies. The method was found to be unsuitable for the effective separation of the process and degradation impurities. It is a UV/Visible test method. It was not suitable.	American Journal of Phytomedicine and Clinical Therapeutics (2013).
2	Determination of the rifamycin antibiotics rifabutin, rifampin, rifapentine and their major metabolites in human plasma via simultaneous extraction coupled with LC/MS/MS.	The method was found to be unsuitable for the effective separation of the process and degradation impurities. It is a Human Plasma test method. It was not suitable.	Journal of Pharmaceutical and Biomedical Analysis (2015).
3	Validation of a simple HPLC‐UV method for rifampicin determination in plasma: Application to the study of rifampicin arteriovenous concentration gradient.	The method was found to be unsuitable for the effective separation of the process and degradation impurities. It is a Human Plasma test method. It was not suitable.	Journal of Pharmaceutical and Biomedical Analysis (2016).
4	Analytical method development and validation of impurity profile in rifapentine.	The method was found to be unsuitable for the effective separation of the process and degradation impurities. The author did not discuss well about the mass balance and degradation studies. In this method, the N‐oxide impurity peak shape is not good. It was not a good stability‐indicating method. It was not suitable.	International Journal of Theoretical & Applied Sciences (2017).
5	Validation and application of a quantitative liquid chromatography tandem mass spectrometry assay for the analysis of rifapentine and 25‐O‐desacetyl rifapentine in human milk.	The method was found to be unsuitable for the effective separation of the process and degradation impurities. It is a Human Plasma test method. It was not suitable.	Journal of Pharmaceutical and Biomedical Analysis (2022).
6	Mitigating matrix effects for LC–MS/MS quantification of nitrosamine impurities in rifampin and rifapentine.	The method was found to be unsuitable for the effective separation of the process and degradation impurities. It is a nitrosamine test method.	Journal of Pharmaceutical and Biomedical Analysis Open (2024).
7	Development and validation of a stability‐indicating high‐performance thin‐layer chromatographic method for estimation of rifapentine in a pharmaceutical formulation	The method was found to be unsuitable for the effective separation of the process and degradation impurities. It is a thin‐layer chromatographic test method. It is not suitable for this target research work.	Separation Science Plus (2024).

*Note:* **The proposed method**: A robust and straightforward reversed‐phase liquid chromatography (RP‐LC) method was developed for the efficient separation and characterization of process‐related and degradation impurities present in the rifapentine drug product. Considering the synthetic pathway of rifapentine, the optimized method effectively achieved baseline separation of all identified impurities. Method development involved the judicious selection of an appropriate stationary phase and fine‐tuning of the mobile phase composition to ensure optimal resolution of analytes with closely related polarities. Furthermore, the method's reliability and stability‐indicating nature were confirmed through extensive forced degradation studies and robustness assessments conducted using analytical quality by design (QbD) principles and software tools. Compared with previously reported analytical methods, this newly established approach offers enhanced selectivity, sensitivity, and superior capability for monitoring the stability and impurity profile of rifapentine formulations.

The robustness of the method was thoroughly assessed using Design Expert software version 13 (Bandaru et al. 2025; Raghupathi et al. 2025; Vemuri et al. 2022), a statistical platform employed to evaluate critical quality attributes (CQAs) and study interactions among key variables. A factorial design approach was applied to systematically examine robustness, thereby supporting the development of a reliable and reproducible liquid chromatography method. Additionally, this QbD‐based strategy contributed to the principles of green chromatography by integrating method optimization with environmental considerations, ensuring both high analytical performance and enhanced sustainability. A robust reversed‐phase liquid chromatography (RP‐HPLC) method was established to effectively separate and characterize four known process‐ and degradation‐related impurities associated with rifapentine. Guided by the synthetic pathway of rifapentine, the method ensured baseline resolution of all targeted impurities. Optimization of chromatographic conditions was achieved through strategic selection of the stationary phase and careful adjustment of the mobile phase composition, enabling efficient separation of structurally and polarity‐related compounds. The method was further supported by extensive forced degradation studies and robustness assessment using an analytical quality by design (QbD) approach, highlighting its reliability and stability‐indicating nature. Compared with previously reported analytical techniques, this method employs a unique strategy and offers superior capability in monitoring rifapentine impurity profiles. Overall, the study delivers a comprehensive and dependable LC method for accurate quantification and control of rifapentine‐related impurities in the final drug product.

## 2 Materials and Methods

### 2.1 Chemicals, Reagents, and Standards

**FIGURE 1:**
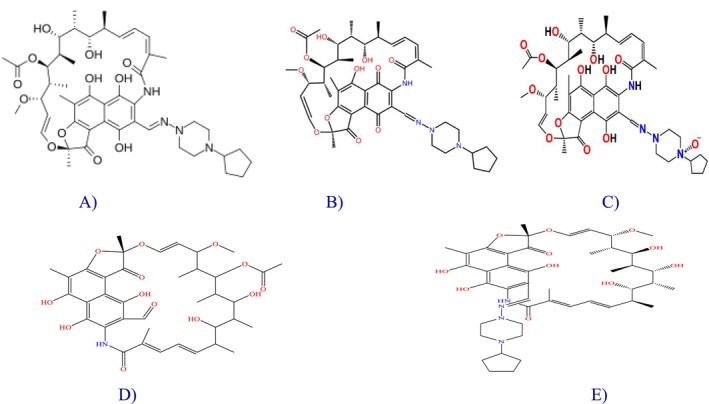
(A) Rifapentine structure. (B) Impurity 1 (process and degradant). (C) Impurity 2 (process and degradant). (D) Impurity 3 (process and degradant). (E) Impurity 4 structure.

High‐purity chemicals were utilized throughout this study to ensure analytical accuracy and reproducibility. Phosphate buffer (KH₂PO₄, Emplura grade), sodium hydroxide (AR grade), acetonitrile (HPLC grade), triethylamine (HPLC grade), orthophosphoric acid (HPLC grade), hydrochloric acid (AR grade), and hydrogen peroxide (H₂O₂, AR grade) were procured from Merck (Mumbai, India). Milli‐Q water (Milli‐Q grade) was used for the preparation of all solutions and during all analytical procedures. Rifapentine working standard (purity 99.8%), along with its known and degradation impurities (Impurity 1*, Impurity 2*, Impurity 3*, and Impurity 4*; Figure 1), was obtained from Pharmaffiliates Private Ltd., Haryana, India. Commercially available rifapentine tablets 150 mg and 300 mg were purchased from a local pharmacy in Hyderabad, Telangana, India.

*Impurity 1: 3‐[[(4‐cyclopentyl‐1‐piperazinyl)imino]methyl]‐rifamycin quinone, (C_47_H_62_N_4_O_12_).

*Impurity 2: 3‐[[(4‐Cyclopentyl‐1‐piperazinyl)imino]methyl]rifamycin N‐Oxide, (C_47_H_64_N_4_O_13_).

*Impurity 3: 3–3‐Formyl rifampicin SV; 1,2‐dihydro‐5,6,9,17,19,21‐hexahydroxy‐23‐methoxy‐2,4,12,16,18,20,22‐heptamethyl‐1,11‐dioxo‐2,7‐(epoxypentadeca[1,11,13]trienimino)naphtho[2,1‐b]furan‐8‐carboxaldehyde 21‐acetate, (C_38_H_47_NO_13_).

*Impurity 4: 3‐[[(4‐Cyclopentyl‐1‐piperazinyl)imino]methyl]‐25‐O‐deacetyl‐rifamycin, (C_45_H_62_N_4_O_11_).

### 2.2 Instrumentations and Software

A reverse‐phase high‐performance liquid chromatography (RP‐HPLC) system (Waters Alliance e2695, Milford, Massachusetts, United States) equipped with a quaternary pump and a photodiode array (PDA) detector was employed for the study. Data acquisition and analysis were performed using Empower 3 software (Waters Corporation, Milford, Massachusetts, United States). Analytical and microbalances (models XP4002S, AX205 Delta Range, and MX5) from Mettler Toledo (Columbus, Ohio, United States) were used for accurate sample weighing. The pH of all solutions was determined using a SevenMulti pH meter (Mettler Toledo, Columbus, Ohio, United States). Sonication of samples was conducted in a Branson 8510 ultrasonic bath (Emerson Electric, St. Louis, Missouri, United States), while forced degradation studies were carried out using a circulating water bath (Precision CIR 19, Thermo Fisher Scientific, Mumbai, India).

### 2.3 Chromatographic Conditions

To establish an efficient separation of rifapentine and its related impurities, a robust reverse‐phase chromatographic method was developed using a YMC Pack C18 column (250 × 4.6 mm, 5 μm particle size). Method optimization was performed to achieve sharp peak shapes and high resolution. The mobile phase was delivered at a flow rate of 1.0 mL/min, and an injection volume of 25 μL was selected to minimize band broadening while maintaining adequate sensitivity. The column temperature was controlled at 30 ± 5 °C to ensure consistent retention times and stable peak responses. UV detection was carried out at 330 nm, which provided optimal sensitivity for rifapentine and its impurities. The total chromatographic run time was 75 min, ensuring sufficient separation of closely eluting components and complete baseline resolution. The optimized gradient program for mobile phase B (%B) was set as follows: 0 min—0%, 3 min—0%, 10 min—25%, 20 min—30%, 25 min—30%, 32 min—40%, 40 min—40%, 45 min—50%, 55 min—70%, 65 min—70%, 70 min—0%, and 75 min—0%. This gradient profile provided effective separation and reproducible chromatographic performance for rifapentine and its associated impurities.

### 2.4 Analytical Solutions

#### 2.4.1 Preparation of Phosphate Buffer Solution

A buffer solution was prepared by dissolving 1.36 g of potassium dihydrogen phosphate (KH₂PO₄) in 1000 mL of water. To this solution, 1 mL of triethylamine was added, and the pH was carefully adjusted to 6.30 using a diluted orthophosphoric acid solution.

#### 2.4.2 Preparation of Mobile Phase A

A mobile phase was prepared by mixing phosphate buffer and acetonitrile in a 90:10 (v/v) ratio. The mixture was then sonicated for 10 min to remove any dissolved gases and ensure a homogeneous solution.

#### 2.4.3 Preparation of Mobile Phase B

Pure acetonitrile (100%) was employed as the solvent.

#### 2.4.4 Preparation of Diluent

The mobile phase B was used as a diluent.

#### 2.4.5 Preparation of Standard Solution (5.0 ppm Concentration)

An accurately weighed quantity of the rifapentine working standard (50.05 mg) was transferred into a 250‐mL volumetric flask. About 120 mL of the diluent was added, and the mixture was sonicated for 5 min to ensure complete dissolution of the analyte. The solution was then diluted to the mark with the same diluent to obtain the stock solution. From this stock solution, a 5.0 mL aliquot was transferred into a 200‐mL volumetric flask and further diluted to volume with the diluent to prepare the final working solution.

#### 2.4.6 Preparation of Rifapentine Drug Substance Sample Solution (1000 ppm Concentration)

An accurately weighed quantity of rifapentine drug substance (50.08 mg) was transferred into a 50‐mL volumetric flask. Approximately 30 mL of diluent was added, and the mixture was sonicated for a few minutes to ensure complete dissolution of the material. The solution was then diluted to volume with the same diluent and mixed thoroughly to obtain a clear and homogeneous solution.

#### 2.4.7 Preparation of Rifapentine Drug Product Sample Solution (1000 ppm Concentration)

An accurately weighed quantity of rifapentine sample powder (250.13 mg, equivalent to 100 mg of rifapentine) was transferred into a 100‐mL volumetric flask. Approximately 60 mL of diluent was added, and the mixture was sonicated for 30 min to ensure complete dissolution of the material. The solution was then diluted to the mark with the same diluent and mixed thoroughly to obtain a clear and homogeneous solution.

#### 2.4.8 Preparation of Placebo Sample Solution (Without Rifapentine API)

An accurately weighed quantity of placebo sample powder (150.09 mg, equivalent to 100 mg of rifapentine) was transferred into a 100‐mL volumetric flask. Approximately 60 mL of diluent was added, and the mixture was sonicated for 30 min to ensure complete dissolution of the material. The solution was then diluted to the mark with the same diluent and mixed thoroughly to obtain a clear and homogeneous solution.

#### 2.4.9 Preparation of Rifapentine‐Impurities Stock Solution

Approximately 5.0 mg of each impurity was accurately weighed and transferred into a 50‐mL volumetric flask. Approximately 30 mL of diluent was added, and the mixture was sonicated for a few minutes to ensure the complete dissolution of impurities. The solution was then diluted to volume with the same diluent and mixed thoroughly to obtain a clear and homogeneous solution (100 ppm).

#### 2.4.10 Preparation of Rifapentine Drug Product Spiked Sample Solution (Each Impurity 0.5% Specification)

**FIGURE 2:**
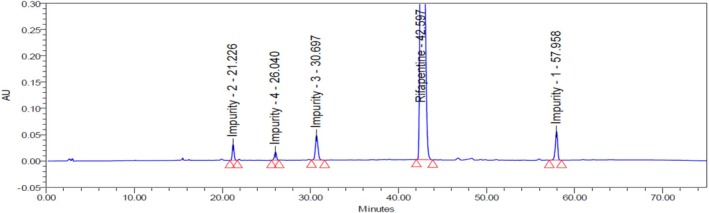
Final optimization test method trial – 5 chromatogram.

An accurately weighed quantity of rifapentine sample powder (250.06 mg, equivalent to 100 mg of rifapentine) was transferred into a 100‐mL volumetric flask. Approximately 60 mL of diluent was added, and the mixture was sonicated for a few minutes to ensure complete dissolution of the material. Subsequently, 5 mL of the impurity stock solution was added to the sample solution, and the volume was made up to the mark with the same diluent. The resulting solution contained each impurity at a concentration of 5.0 ppm (Figure 2).

## 3 Results and Discussion

### 3.1 Optimization of Chromatographic Conditions

The present research aims to develop a precise, accurate, specific, linear, and robust liquid chromatography (LC) method for the estimation of process‐related and degradation impurities in the rifapentine drug product. Furthermore, a comprehensive risk assessment is performed using the analytical quality by design (AQbD) approach to ensure method reliability and to identify critical parameters influencing the analytical performance. This systematic approach facilitates the development of a scientifically sound and regulatory‐compliant method suitable for routine quality control and stability testing of rifapentine formulations.

During the initial phase of method development, comprehensive information was gathered to enable a systematic and scientifically sound analytical approach. Detailed data regarding the physicochemical and stability characteristics of rifapentine, as documented in the approved New Drug Application (NDA), were thoroughly reviewed. Rifapentine is a weakly basic compound (pKa ≈ 7.9) due to the presence of an imine functional group and exhibits a melting point in the range of approximately 180–185 °C, at which it decomposes. It is practically insoluble in water but freely soluble in organic solvents such as chloroform, methanol, dimethyl sulfoxide (DMSO), and acetone. The drug substance is light‐sensitive and demonstrates instability under acidic and strongly basic conditions, whereas it remains relatively stable in neutral to slightly alkaline media (pH 7–8). Rifapentine undergoes degradation in acidic environments, indicating susceptibility to gastric conditions. The recommended storage condition is at 25 °C, protected from light and moisture, to maintain physicochemical stability. Furthermore, rifapentine exhibits strong UV absorption due to its extended conjugated chromophore system, making UV–Visible detection a suitable and selective analytical choice for quantitative determination. This comprehensive understanding of the compound's intrinsic properties facilitated the rational design and development of a robust and reliable analytical strategy. Based on the comprehensive physicochemical and literature information gathered during the preliminary phase, a systematic approach was adopted to select the most appropriate chromatographic conditions for the initial method development trials. The selection of the mobile phase and stationary phase was guided by the solubility characteristics, polarity, and stability profile of rifapentine, as well as its chromatographic behavior reported in the literature. Various combinations of organic solvents and aqueous buffers were evaluated to achieve optimal resolution, peak symmetry, and reproducibility. Similarly, different stationary phases were screened to ensure adequate retention and separation efficiency, taking into account the compound's hydrophobic and ionizable nature. The finalized chromatographic parameters, including the choice of column, mobile phase composition, flow rate, detection wavelength, and injection volume, were systematically optimized to obtain a robust and reproducible method. A comprehensive summary of all development trials, including the specific chromatographic conditions employed, as well as their respective results & observations, is presented in (Table 2 and Figures S1–S4). During the course of method development trials, several critical challenges were encountered, including poor peak purity and distorted peak shapes, interference from unknown peaks, inadequate separation between degradation products and unidentified impurities, and incomplete recovery of degradation impurities due to the inappropriate selection of the diluent. These issues were systematically addressed through the application of scientific rationale, a comprehensive understanding of chromatographic principles, and an informed selection of stationary and mobile phase components based on polarity and column chemistry. Consequently, an optimized chromatographic method was established, achieving clear and reproducible separation of all process‐ and degradation‐related impurities with satisfactory system suitability results. The finalized test method is proposed for validation in accordance with ICH Q2(R2) and USP <1225> guidelines to ensure its accuracy, precision, specificity, and robustness. Furthermore, to enhance the reliability and overall performance of the LC method, a robustness study should be conducted following the AQbD framework, facilitating the identification of critical method parameters (CMPs) and CQAs that govern method consistency and performance.

**TABLE 2:** Optimization of chromatographic conditions.

No. of trials	Method details	Column details	Results and observations	Method status
Trial 1	Mobile phase—A: phosphate buffer (pH 4.0)/acetonitrile (90:10 v/v); B: water/acetonitrile (40:60 v/v), set a linear gradient and a runtime of 75 min. Flow rate: 1.0 mL/min, injection volume: 25 μL, UV: 330 nm.	Inert Sustain C18 (150 × 4.6) 3 μm, column temperature: 25 °C.	Impurity 1 and unknown imp both were merged. Baseline is also not good. One unknown imp was interference at imp–3. Impurities 2 and 4 both were eluted closely. The chromatogram can be seen in the Figure S1. The method details were not suitable.	Rejected
Trial 2	Mobile phase—A: phosphate buffer (pH 4.5)/acetonitrile (80:20 v/v); B: water/acetonitrile (30:70 v/v), set a linear gradient and a runtime of 70 min. Flow rate: 1.0 mL/min, injection volume: 25 μL, UV: 330 nm.	X Bridge C18 (150 × 4.6) 3.5 μm, column temperature: 25 °C.	Impurity 1 peak shape was not good. Impurity 3 peak purity was not passed. All impurity peaks were separated well. The chromatogram can be seen in the Figure S2. The method details were not suitable.	Rejected
Trial 3	Mobile phase—A: KH_2_PO_4_ phosphate buffer (pH 5.0)/acetonitrile (80:20 v/v); B: water/acetonitrile (30:70 v/v), set a linear gradient and a runtime of 70 min. Flow rate: 1.0 mL/min, injection volume: 25 μL, UV: 330 nm.	X Bridge C18 (150 × 4.6) 3.5 μm, column temperature: 25 °C.	Impurity 3 and unknown imp both were merged. One unknown imp and rifapentine main peak both were merged. Impurity 3 was poor peak purity. The chromatogram can be seen in the Figure S3 . The method details were not suitable.	Rejected
Trial 4	Mobile phase—A: KH_2_PO_4_ phosphate buffer (pH 5.5)/acetonitrile (70:30 v/v); B: 100% acetonitrile, set a linear gradient and a runtime of 70 min. Flow rate: 1.0 mL/min, injection volume: 25 μL, UV: 330 nm.	YMC Pack C18 column (250 × 4.6) mm, 5 μm, column temperature: 30 °C.	Impurity 1 and unknown imp both were merged. Impurities 3 and 4 both were merged. Impurity 2 peak shape was not good. The chromatogram can be seen in the Figure S4 . The method details were not suitable.	Rejected
Trial 5	Mobile phase—A: KH_2_PO_4_ phosphate buffer (pH 6.3)/acetonitrile (90:10 v/v); B: 100% acetonitrile, set a linear gradient and a runtime of 75 min. Flow rate: 1.0 mL/min, injection volume: 25 μL, UV: 330 nm.	YMC Pack C18 column (250 × 4.6) mm, 5 μm, column temperature: 30 °C.	A resolution exceeding 2.0 was successfully achieved between rifapentine and all related impurities, indicating good separation. The system's suitability results were found to be satisfactory. Baseline is good. The spiked chromatogram can be seen in Figure 2. The method conditions were found to be satisfactory and deemed suitable to proceed to the next step. This method should be verified through validation studies in accordance with ICH Q2 (R2) guidelines.	Approved

#### 3.1.1 Analytical Target Profile

The developed analytical method was meticulously optimized to ensure a resolution greater than 2.0 between rifapentine and all its related impurities, thereby providing effective and distinct separation of critical components. Further refinement of the method enabled precise quantification of degradation products and related impurities across a broad concentration range, from the reporting threshold of 0.03% up to 150% of the working concentration (equivalent to 0.5% of the specification level). The method performance criteria were established to demonstrate acceptable accuracy, with recovery values maintained within 70%–130%, and repeatability confirmed by a relative standard deviation (RSD) of less than 5.0%. Collectively, these validation parameters substantiate the robustness and reliability of the method for comprehensive impurity profiling and stability evaluation of rifapentine and its associated substances.

#### 3.1.2 Risk Identification

The risk assessment process commenced with the systematic identification of potential variables that could impact the analytical method's performance. A comprehensive and structured approach was adopted to assess all possible sources of variation, including instrumental conditions, environmental influences, sample properties, and analyst‐related factors. To support this evaluation, an Ishikawa (fishbone) diagram was employed as an effective quality risk management tool, providing a visual representation and categorization of factors that may affect method robustness (Figure S5). This thorough assessment enabled the recognition of critical method parameters that necessitate careful monitoring and stringent control throughout method development and validation to ensure consistent and reliable analytical performance.

### 3.2 Method Validation

Analytical method validation is a structured and scientifically driven process designed to confirm that an analytical procedure is fit for its intended use. It provides documented assurance that the method consistently delivers accurate, precise, and reliable results within predefined criteria. In the pharmaceutical field, method validation plays a vital role in ensuring data integrity, supporting quality assurance, and maintaining regulatory compliance throughout drug development and manufacturing.

For rifapentine and its related impurities, method validation serves to verify that the developed analytical procedure can accurately detect and quantify both identified, unidentified, and degradation impurities in the presence of the active pharmaceutical ingredient (API). The validation study evaluates essential performance attributes, including specificity, linearity, accuracy, precision, limits of detection and quantification, robustness, and solution stability. Compliance with ICH Q2(R2) (ICH 2023) and applicable regulatory standards ensures that the validated method is scientifically robust, reliable, and appropriate for routine quality control as well as stability assessment of rifapentine drug products.

#### 3.2.1 System Suitability Test

To verify the performance and reliability of the chromatographic system, both the standard and system suitability solutions were prepared in accordance with the established test procedure and injected into the system. The key system suitability parameters, such as retention time, theoretical plate count, tailing factor, and percentage relative standard deviation (%RSD) of replicate injections, were thoroughly assessed. All evaluated parameters complied with the predefined acceptance limits, thereby confirming that the chromatographic system was adequate and suitable for the analysis of rifapentine and its related substances.

#### 3.2.2 Specificity (Blank and Interference of Impurities)

**FIGURE 3:**
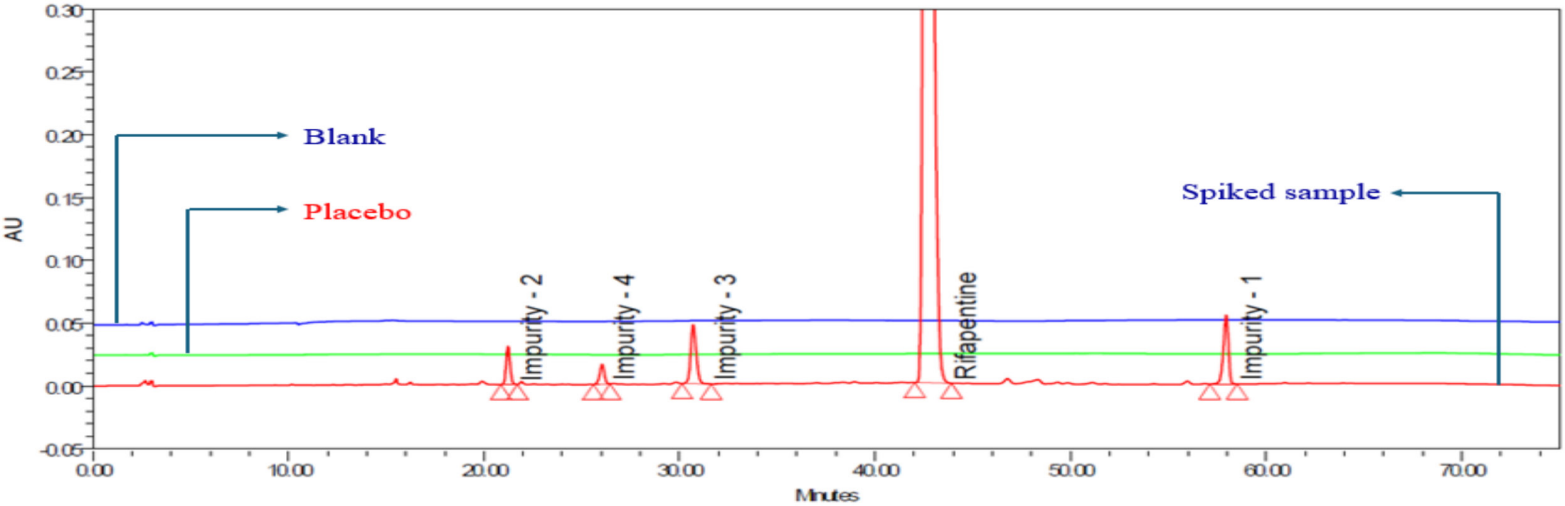
Overlay chromatogram of blank, placebo, and spiked sample in the specificity test.

To evaluate the specificity of the developed method, a series of solutions, including a blank, individual known impurity solutions, and a spiked sample containing 0.5% of known impurities (Impurities 1, 2, 3, and 4) (ICH 2006), were prepared and injected into the LC system. The chromatographic analysis revealed that the rifapentine peak and all impurity peaks were well separated from one another, demonstrating excellent chromatographic resolution. No interference from the blank and placebo was detected at the retention times corresponding to either rifapentine or its known impurities, confirming the absence of co‐eluting substances. Additionally, the purity angle values for rifapentine and each impurity in the spiked sample were found to be lower than their respective purity thresholds, indicating spectral homogeneity and confirming peak purity. These results collectively demonstrate that the developed method is specific for the determination of rifapentine and its impurities (Figure 3). The detailed outcomes are summarized in Table 3.

**TABLE 3:** Forced degradation study results.

Impurity name	RRT	As such a sample	Thermal deg. sample	Humidity deg. sample	Photo‐exposed solid sample	Acid deg. sample	Base deg. sample	Oxidation deg. sample
			105 °C/2 days	80% RH/2 days	1.2 mill. lux h/200 W per m^2^	2 N HCl/1 h	0.2 N NaOH/1 h	30% H_2_O_2_/1 h
Imp‐2 (purity flag)	**0.500**	0.10	5.38	0.20	0.44	0.11	0.13	0.11
	**NA**	NA	Purity angle: 0.214; purity threshold: 0.351.	NA	NA	NA	NA	NA
Imp‐4	**0.611**	ND	0.29	ND	ND	0.03	ND	ND
Imp‐3 (purity flag)	**0.720**	0.12	1.46	0.66	0.42	7.88	0.51	0.48
	**NA**	NA	NA	NA	NA	Purity angle: 0.223; purity threshold: 0.386	NA	NA
Imp‐1 (purity flag)	**1.360**	0.18	4.42	0.78	1.00	0.14	0.55	0.52
	**NA**	NA	Purity angle: 0.314; purity threshold: 0.428.	NA	NA	NA	Purity angle: 0.285; purity threshold: 0.392.	Purity angle: 0.318; purity threshold: 0.562.
Rifapentine (purity flag)	**NA**	NA	Purity angle: 0.243; purity threshold: 0.534.	Purity angle: 0.384; purity threshold: 0.713.	Purity angle: 0.312; purity threshold: 0.472.	Purity angle: 0.347; purity threshold: 0.634.	Purity angle: 0.453; purity threshold: 0.685.	Purity angle: 0.374; purity threshold: 0.641.
**Major unspecified impurity**		0.12	0.85	0.11	0.12	0.49	0.13	0.14
**Total impurities:**		0.52	11.54	1.64	1.86	8.65	1.32	1.25
**Assay by HPLC:**		**99.4**	**87.14**	**98.23**	**97.9**	**90.34**	**98.2**	**98.6**
**% Degradation:**		**NA**	**11.02**	**1.12**	**1.34**	**8.13**	**0.8**	**0.73**
**% Mass balance:**		**NA**	**98.2**	**99.4**	**99.2**	**98.5**	**99.0**	**99.3**

#### 3.2.3 Specificity (Forced Degradation Studies)

Forced degradation studies play a vital role in the analytical development of rifapentine drug substances and drug products. These studies aim to evaluate the inherent stability of the drug molecule when exposed to various stress conditions, such as acidic, basic, oxidative, thermal, and photolytic environments. By deliberately subjecting rifapentine to these stressors, potential degradation pathways and products can be identified, allowing a deeper understanding of the molecule's chemical behavior and confirming the stability‐indicating nature of the analytical method, particularly HPLC (Blessy et al. 2014; ICH 1996; Campbell et al. 2024; Kronsbein et al. 2025; Konduru et al. 2020; Vishnu Murthy, Krishnaiah, Kumar, and Mukkanti 2013; Vishnu Murthy et al. 2011; Vishnu Murthy, Krishnaiah, Srinivas, et al. 2013). The insights gained from forced degradation studies are crucial for determining suitable storage conditions, selecting suitable packaging materials, and assessing the product's shelf life, thereby ensuring its safety and efficacy throughout its intended use. Furthermore, a comprehensive understanding of rifapentine's degradation profile strengthens regulatory submissions by clearly demonstrating that the developed analytical method can effectively differentiate the active pharmaceutical ingredient from its degradation‐related impurities. The method achieved satisfactory mass balance results within the established acceptance criteria of 98.0%–102.0% across all stress conditions. Based on the observed chemical and physical degradation behavior, the method is confirmed to be stability‐indicating in nature. Owing to its robustness, specificity, and regulatory compliance, this method is suitable for routine quality control testing as well as analytical research and development activities in regulatory‐approved laboratories.

##### 3.2.3.1 Acid Hydrolysis Study

**FIGURE 4:**
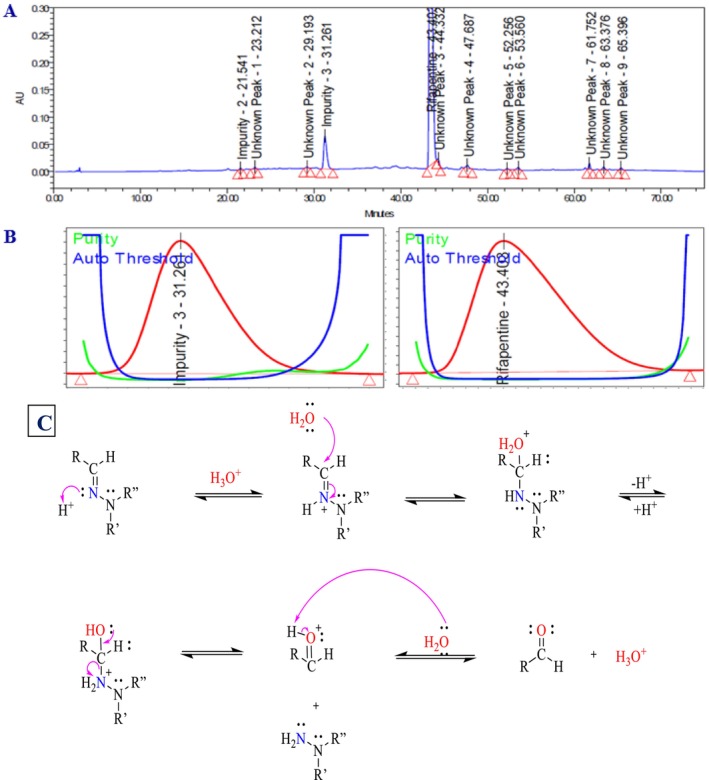
(A) Acid degradation chromatogram. (B) Rifapentine and impurity 3 (degradation product) peak purity spectra. (C) Acid degradation reaction mechanism between rifapentine and impurity 3 (major degradant).

The acid degradation study of the rifapentine drug product was performed to assess its stability under acidic stress conditions. A precisely weighed quantity of rifapentine powder (250.09 mg, equivalent to 100 mg of rifapentine) was placed in a 100‐mL volumetric flask, to which 5 mL of 2 N hydrochloric acid solution was added. The mixture was maintained in a water bath at 50 °C for 1 h to facilitate complete degradation. After cooling, 5 mL of 2 N sodium hydroxide solution was introduced to neutralize the reaction mixture. The solution was then diluted with approximately 50 mL of diluent, sonicated for 30 min to ensure complete dissolution, and finally made up to volume with the same diluent. The sample prepared was subsequently injected into the LC system for analysis. The representative chromatogram, peak purity index, reaction mechanism, and related data are presented in Figure 4A–C and Table 3. The results demonstrated that rifapentine is highly susceptible to acid hydrolysis, with Impurity 3 identified as a major degradation product, confirming the compound's sensitivity under acidic conditions.

##### 3.2.3.2 Base Hydrolysis Study

The base‐induced degradation study of the rifapentine drug product was conducted to evaluate its stability under alkaline stress conditions. An accurately weighed portion of rifapentine powder (250.16 mg, equivalent to 100 mg of the active drug) was transferred into a 100‐mL volumetric flask. To this, 5 mL of 0.2 N sodium hydroxide solution was added, and the mixture was maintained at ambient temperature for 1 h to allow complete degradation. After the reaction period, the mixture was cooled to room temperature, followed by the addition of 5 mL of 0.2 N hydrochloric acid to neutralize the excess alkali. Subsequently, approximately 50 mL of diluent was added, and the solution was sonicated for 30 min to ensure complete dissolution before being diluted to volume with the same diluent. The resulting solution was then injected into the LC system for analysis.

**FIGURE 5:**
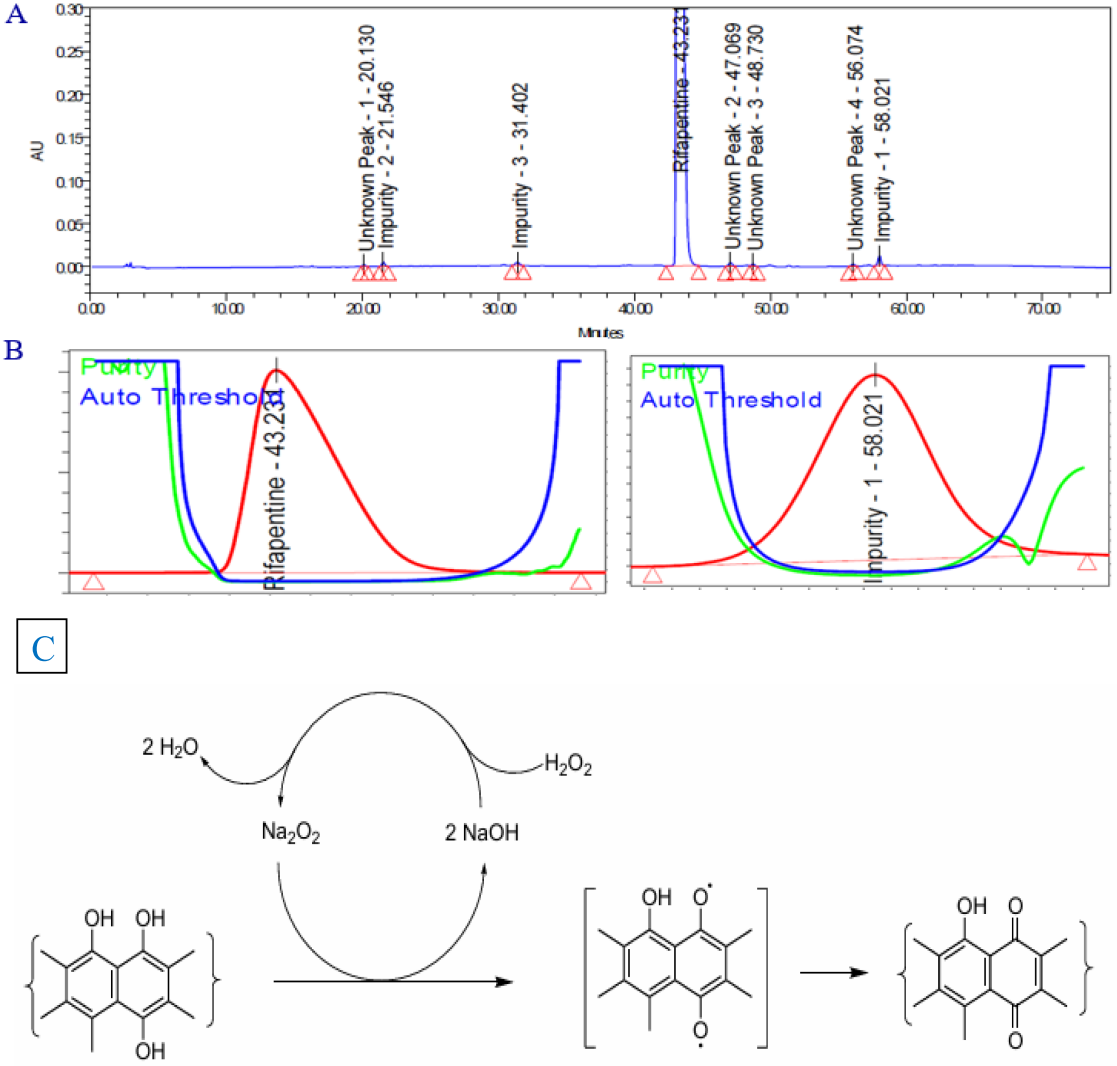
(A) Base degradation chromatogram. (B) Rifapentine and impurity 1 (degradation product) peak purity spectra. (C) Degradation pathway between rifapentine and impurity 1.

The chromatographic data, including the representative chromatogram, peak purity index, and degradation pathway, are illustrated in Figure 5A–C and summarized in Table 3. The study findings revealed that rifapentine undergoes significant degradation under basic conditions, forming Impurity 1 as the principal degradation product. These results confirm that rifapentine is highly susceptible to hydrolysis in alkaline environments.

##### 3.2.3.3 Peroxide (Oxidation) Degradation Study

The oxidative degradation study of the rifapentine drug product was performed to assess its stability under peroxide‐induced stress conditions. A precisely weighed amount of rifapentine powder (250.21 mg, equivalent to 100 mg of the active pharmaceutical ingredient) was transferred into a 100‐mL volumetric flask. To this, 5 mL of 30% hydrogen peroxide (H₂O₂) solution was added, and the mixture was kept at room temperature for 1 h to promote oxidative degradation. After the designated period, the reaction mixture was allowed to cool to ambient temperature. Subsequently, about 50 mL of diluent was added, and the solution was sonicated for 30 min to ensure complete dissolution, followed by dilution to volume with the same diluent. The sample prepared was then subjected to LC analysis.

**FIGURE 6:**
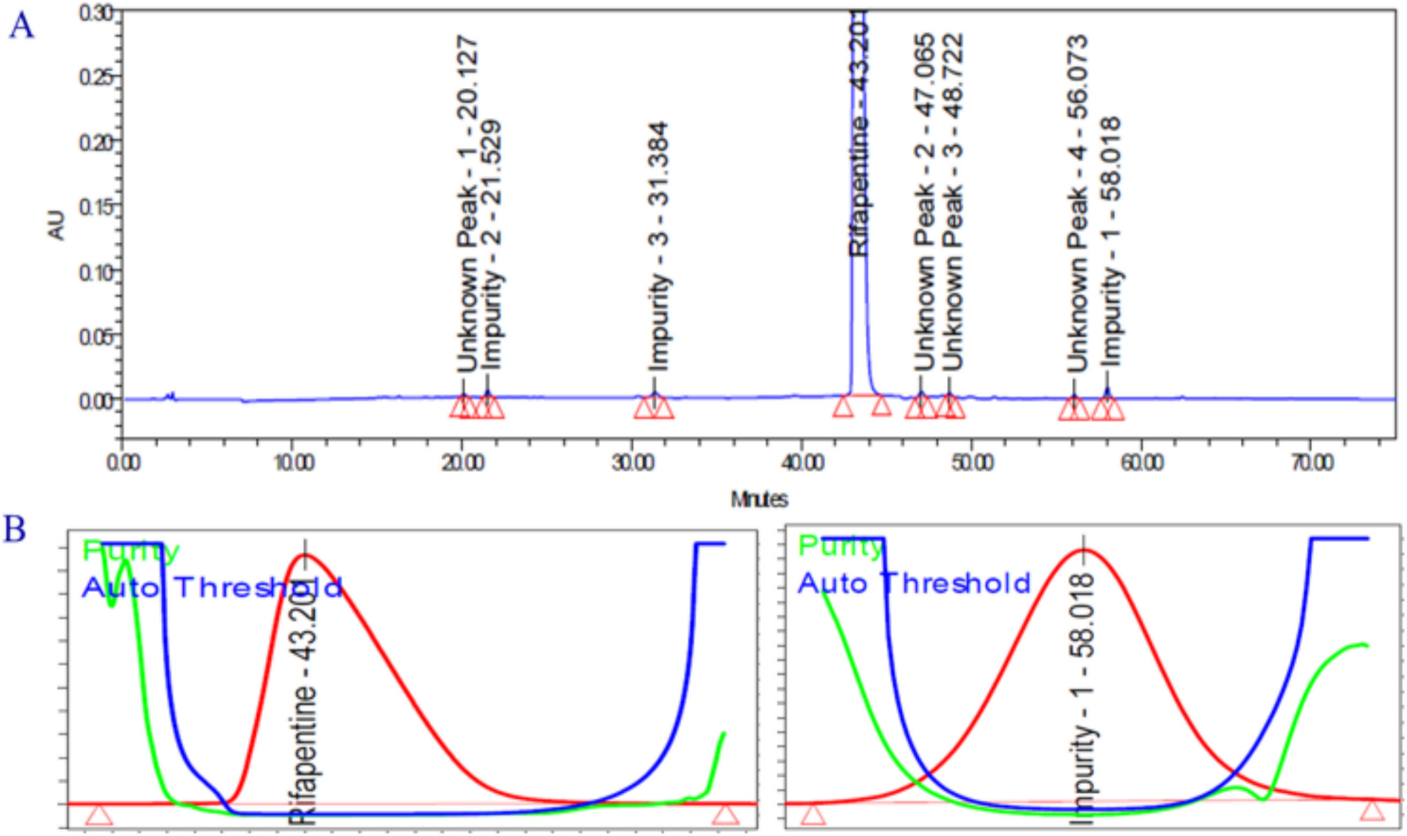
A) Peroxide degradation chromatogram, B) Rifapentine and Impurity 1 (Degradation product) Peak purity spectra's.

The chromatographic results, including the representative chromatogram, peak purity data, and proposed degradation mechanism, are presented in Figure 6A,B and summarized in Table 3. The results indicated that rifapentine is highly prone to oxidative degradation, with Impurity 1 identified as the predominant degradation product. These findings demonstrate that rifapentine is particularly sensitive to oxidative stress conditions.

##### 3.2.3.4 Thermal Degradation Study (Effect of Temperature)

**FIGURE 7:**
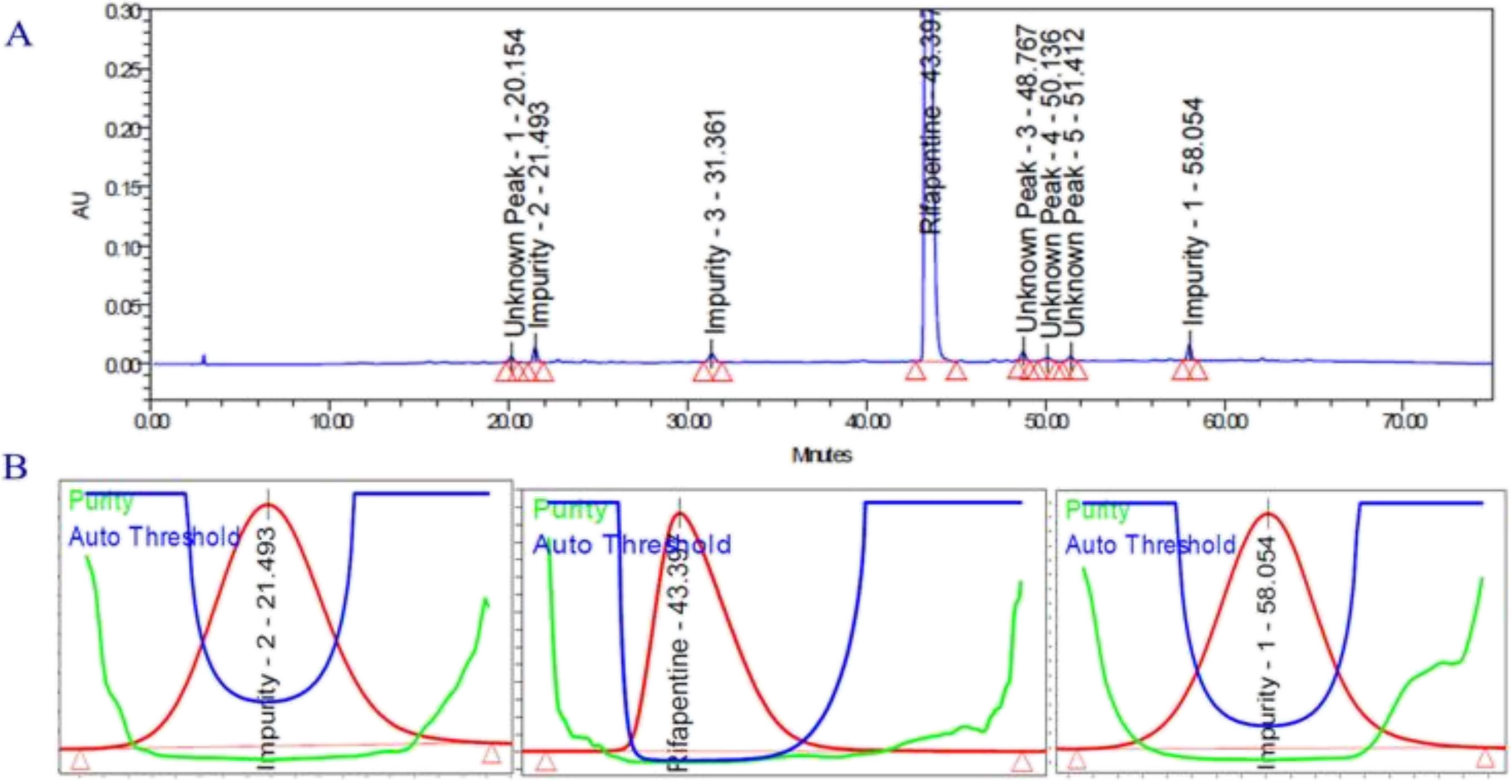
(A) Thermal degradation chromatogram. (B) Rifapentine and Impurity 2 and 1 (degradation products) peak purity spectra.

A thermal degradation study was performed on the rifapentine drug product to assess its stability under elevated temperature conditions. The sample was exposed to heat in a controlled oven maintained at 105 °C for a duration of 2 days. Following the exposure period, an accurately weighed portion of the thermally stressed sample (250.24 mg) was transferred into a 100‐mL volumetric flask. About 50 mL of the diluent was added, and the mixture was sonicated for 30 min to achieve complete dissolution, after which the volume was made up with the same diluent. The prepared solution was then analyzed using the optimized chromatographic method. The corresponding chromatograms and data are provided in Figure 7A,B and Table 3. The analysis revealed a significant degradation of rifapentine upon heating, with Impurity 2 and Impurity 1 identified as the major degradation products. These results confirm that rifapentine exhibits pronounced sensitivity to elevated thermal conditions.

##### 3.2.3.5 Humidity Degradation Study (Effect of Moisture)

The humidity‐induced degradation study of the rifapentine drug product was carried out to assess its stability under high‐moisture conditions. The sample was exposed to 80% relative humidity (RH) in a controlled desiccator for 7 days to simulate humid environmental stress. Following the exposure period, an accurately weighed quantity of the stressed sample (250.21 mg) was transferred into a 100‐mL volumetric flask. About 50 mL of diluent was added, and the mixture was sonicated for 30 min to achieve complete dissolution. The volume was then made up to the mark with the same diluent. The prepared solution was analyzed using the validated chromatographic method. The corresponding chromatograms and analytical data are provided in Table 3. The results demonstrated that rifapentine showed no significant degradation under high‐humidity conditions, indicating that the drug product exhibits good stability when subjected to moisture stress.

##### 3.2.3.6 Photolytic Degradation Study (Effect of Light)

**FIGURE 8:**
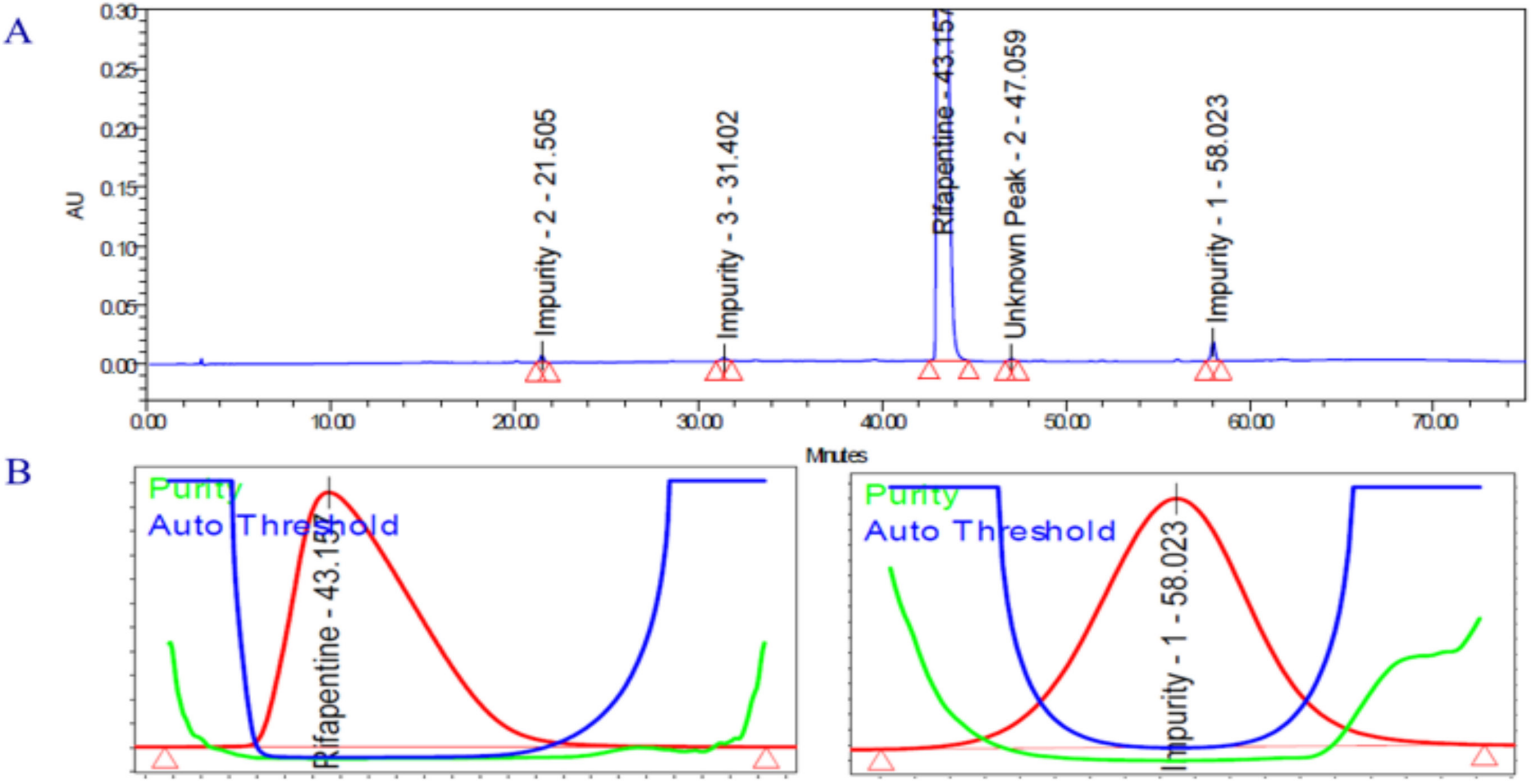
(A) Photolytic degradation chromatogram. (B) Rifapentine and Impurity 1 (degradation products) peak purity spectra.

The photostability study of the rifapentine drug product was conducted in compliance with ICH Q1B guidelines to evaluate its susceptibility to light exposure. The sample was subjected to controlled light conditions, simulating photolytic stress, to determine any potential degradation. After the exposure period, an accurately weighed portion of the stressed sample (250.18 mg) was transferred into a 100‐mL volumetric flask. Approximately 50 mL of diluent was added, and the solution was sonicated for 30 min to ensure complete dissolution before making up the volume with the same diluent. The resulting solution was analyzed using the validated chromatographic method. The chromatograms and corresponding data are presented in Figure 8A,B and Table 3. The analytical results indicated that rifapentine did not undergo significant degradation under photolytic stress, demonstrating that the drug product is stable when exposed to UV light conditions.

#### 3.2.4 Precision Study

The precision of the developed analytical method was assessed through repeatability and intermediate precision (ruggedness) studies. Method precision was evaluated using one freshly prepared Unspiked sample and six test samples spiked with Impurities 1, 2, 3, and 4 at the specification level of 0.5%, following the validated analytical procedure. Repeatability was examined by analyzing six freshly prepared drug solutions, each containing 50 ppm of the individual known impurities, under identical experimental conditions on the same day. Intermediate precision was determined by analyzing six independently prepared sample solutions of the same concentration on different days, employing different LC instruments and chromatographic column batches. The %RSD for all impurities was observed to be below 5.0%, confirming that the method exhibits excellent precision and reproducibility under varying laboratory conditions. These findings demonstrate the reliability of the method developed for routine quality analysis. The representative chromatograms and corresponding quantitative results are shown in Table 4.

**TABLE 4:** Method validation results.

S.No.	Parameter	Imp‐2	Imp‐4	Imp‐3	Imp‐1
1	Specificity (peak purity)	Pass	Pass	Pass	Pass
	Limit of detection and quantitation				
	LOD (% w/w)	0.01% (0.01 μg/mL)	0.01% (0.01 μg/mL)	0.01% (0.01 μg/mL)	0.01% (0.01 μg/mL)
	S/N ratio	3.9	3.4	4.1	3.7
	LOQ (% w/w)	0.03% (0.301 μg/mL)	0.03% (0.302 μg/mL)	0.03% (0.300 μg/mL)	0.03% (0.302 μg/mL)
	S/N ratio	12.1	11.8	12.4	12.3
	LOQ precision (%RSD)	5.6	6.3	5.8	7.4
	Linearity				
	Slope of the regression line	7283.10	4840.46	8532.45	8546.49
	Y‐Intercept	406.22	227.16	209.06	425.69
	% Y‐Intercept (≤ 5.0%)	1.12	0.91	0.49	0.96
	Regression coefficient (*R* ^2^)	0.9997	0.9998	0.9996	0.9992
	Precision (% RSD)				
	Method precision	1.12	1.84	1.29	1.62
	Intermediate precision	1.26	1.62	1.35	1.58
	Accuracy (% recovery)				
	At 50% level	99.4	101.2	101.8	102.8
	At 100% level	99.2	100.3	101.6	98.9
	At 150% level	98.9	99.1	102.3	99.7
	Solution stability (benchtop) spiked sample solution (% of results variation) limits: ± 0.1%				
	24 h	0.03	0.02	0.04	0.01
	36 h	0.05	0.04	0.06	0.03
	48 h	0.08	0.07	0.09	0.05
	Solution stability (refrigerator) 2 ~ 8 °C spiked sample solution (% of results variation) limits: ± 0.1%				
	24 h	0.02	0.01	0.02	0.01
	36 h	0.03	0.02	0.04	0.02
	48 h	0.04	0.04	0.06	0.03

#### 3.2.5 Linearity

**FIGURE 9:**
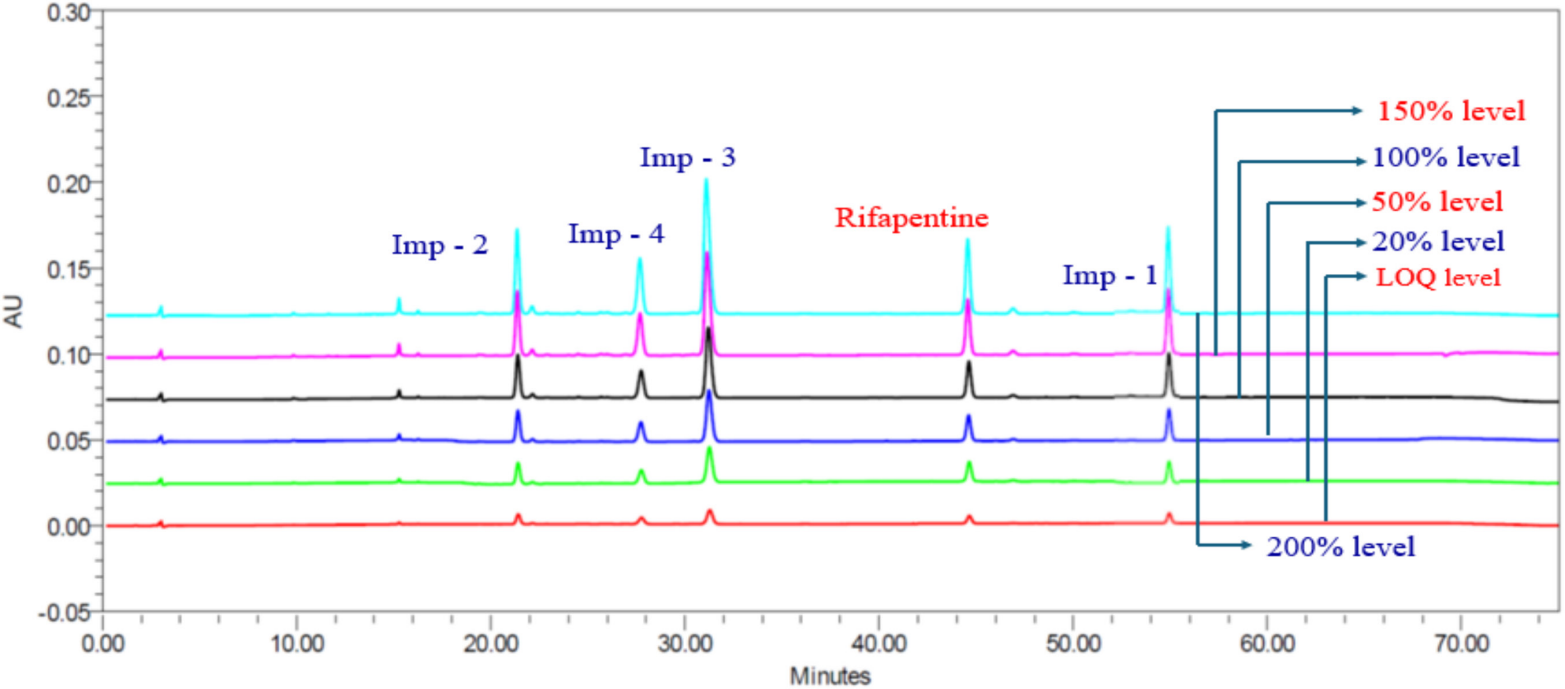
Linearity study overlay chromatogram.

The linearity of the detector response for rifapentine and its specified degradation impurities was assessed by preparing standard solutions at eight different concentration levels, corresponding to the limit of quantitation (LOQ), 20%, 50%, 100%, 150%, and 200% of the target specification level relative to the test concentration. Each concentration level was analyzed following the validated analytical procedure. Calibration curves were plotted by correlating the peak area responses with their respective analyte concentrations. From these curves, the regression coefficients (*R* ^2^) and percentage Y‐intercepts at the 100% response level were calculated for rifapentine‐related impurities. All correlation coefficients were found to exceed the predefined acceptance criteria, while the percentage Y‐intercepts remained within acceptable limits, confirming excellent linearity of the method over the studied concentration range. The slope and intercept values of the calibration plots are summarized in Table 4, and representative chromatograms are presented in Figure 9.

#### 3.2.6 Accuracy

The accuracy of the developed analytical method was evaluated through recovery experiments by spiking known impurities of rifapentine into the test sample at concentration levels ranging from 50% to 150% of the specified limit. Additionally, the recovery of the unspecified impurity (rifapentine) was assessed by spiking it into the drug substance solution over the same concentration range. To ensure consistency and reproducibility, three replicate preparations were analyzed at both the 50% and 150% levels. All spiked samples were injected into the LC system, and the percentage recovery for rifapentine and its specified related impurities was calculated at each concentration level. The mean recovery values for all components were within the predefined acceptance criteria, confirming the high accuracy and reliability of the developed method. The recovery data are summarized in Table 4, and representative chromatograms are provided in Figure S6.

#### 3.2.7 LOD and LOQ

The limit of detection (LOD) and LOQ for rifapentine and its known impurities (Impurities 1, 2, 3, and 4) were established using the signal‐to‐noise (S/N) ratio method. Standard solutions of rifapentine spiked with the respective impurities were prepared in the appropriate diluent and analyzed under the optimized chromatographic conditions. The S/N ratios for each analyte were measured, and the LOD and LOQ were determined based on the criteria of S/*N* ≥ 3 for detection and S/*N* ≥ 10 for quantitation. All obtained values met the acceptance requirements outlined in regulatory guidelines, confirming that the developed method possesses sufficient sensitivity for the reliable detection and quantification of rifapentine and its related impurities. The summarized results are provided in Table 4, and representative chromatograms are displayed in Figure S7.

#### 3.2.8 Precision at the LOQ

The precision of the method at the LOQ level was assessed by preparing six individual sample solutions containing rifapentine and its related impurities (Impurities 1, 2, 3, and 4) at concentrations corresponding to their respective LOQ values. Each prepared solution was injected once into the liquid chromatographic system under identical analytical conditions. The precision was evaluated by calculating the %RSD of the peak areas obtained for each impurity across the six replicate analyses. The %RSD values for all impurities were found to be within the established acceptance criteria, confirming that the method demonstrates satisfactory precision at the LOQ level. A summary of these results is provided in Table 4.

#### 3.2.9 Solution Stability

The stability of the standard and sample solutions of rifapentine and its related impurities was assessed under both refrigerated and ambient storage conditions to verify the reliability of analytical measurements over time. Standard solutions and impurity‐spiked sample solutions were stored at 2 °C, 8 °C, and room temperature (25 °C) and analyzed at predetermined time intervals using the same chromatographic conditions. The study results indicated that both the standard and sample solutions remained stable for up to 48 h under all tested conditions, with no notable variations in peak area responses or impurity profiles. These findings confirm that the prepared solutions are stable for at least 48 h, ensuring the accuracy and consistency of the analytical method. The detailed results of the solution stability study are presented in Table 4.

#### 3.2.10 Robustness and AQbD Study

**FIGURE 10:**
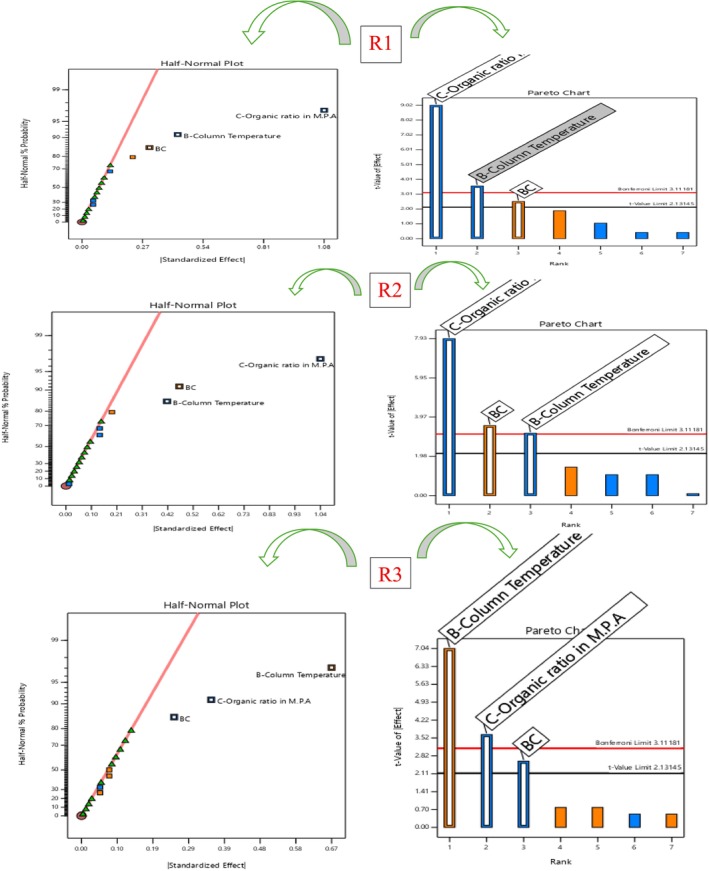
Half‐normal and Pareto charts for R1, R2, and R3 responses.

**FIGURE 11:**
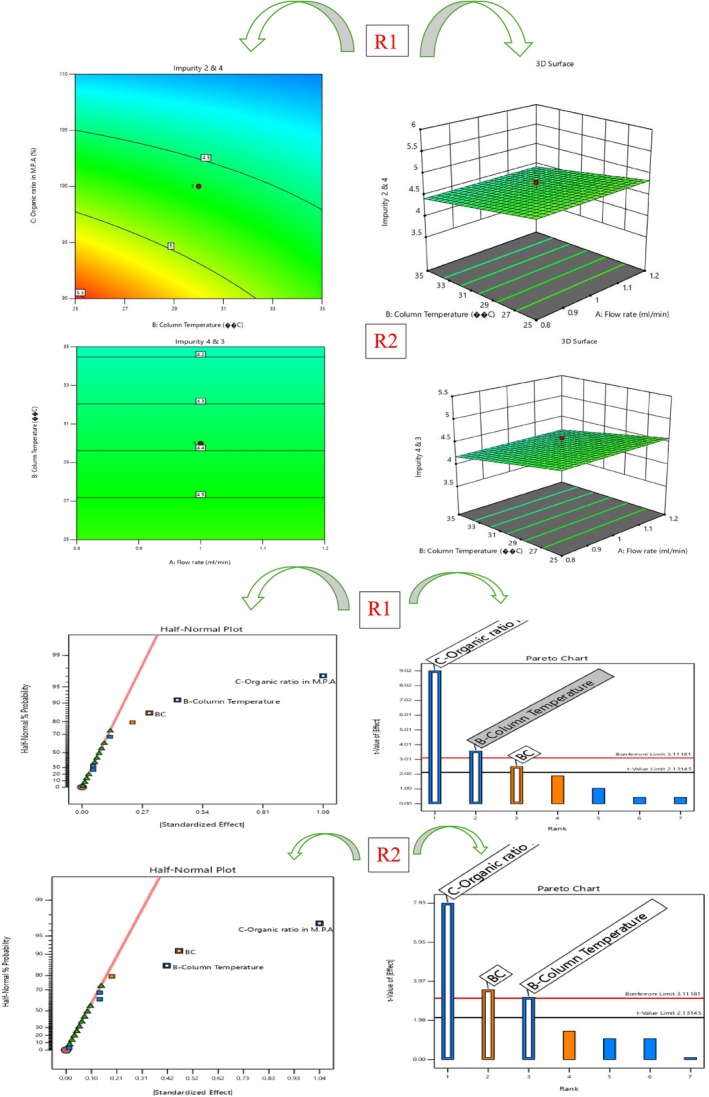
Contour plot and 3D surface plot for R1, R2, and R3 responses.

**FIGURE 12:**
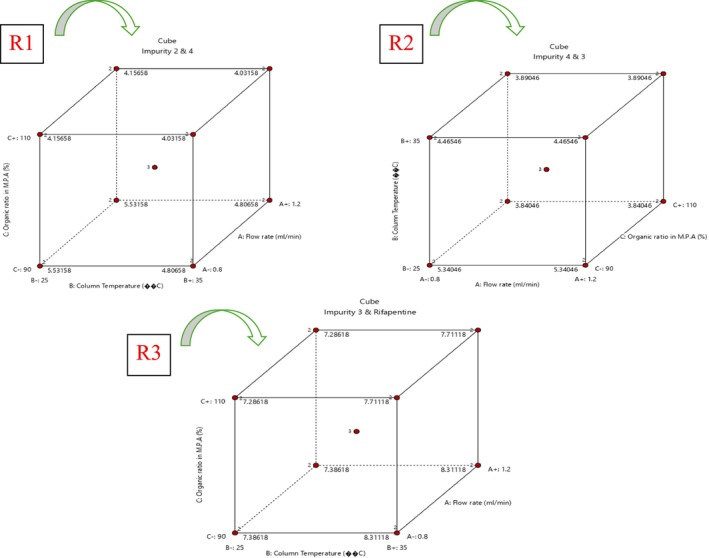
3D cube plot for R1, R2, and R3 responses.

In alignment with the AQbD framework, a robustness evaluation was conducted to verify the consistency and reliability of the developed chromatographic method when subjected to minor, intentional variations in CMPs (Ettaboina et al. 2025; Katakam et al. 2021; Muchakayala et al. 2022; Nathi et al. 2023; Subramanian et al. 2020). The objective of this study was to ensure that small, routine fluctuations in analytical conditions would not significantly impact method performance. A QbD‐driven design of experiments (DoE) approach was employed using Design Expert software to systematically study the effects of the selected CMPs. Based on a preliminary risk assessment, three key parameters were identified: flow rate (1.0 ± 0.2 mL/min), organic solvent ratio in mobile phase A (100 mL ± 10%), and column temperature (30 ± 5 °C). A three‐factor experimental design, consisting of 19 runs with three center points, zero blocks, and two replicates, was performed using spiked sample solutions. The study focused on evaluating three responses: R1 (resolution between Impurity 2 and Impurity 4), R2 (resolution between Impurity 4 and Impurity 3), and R3 (resolution between Impurity 3 and rifapentine) to determine the impact of the CMPs on chromatographic separation. Results from the analysis of variance (ANOVA), summarized in Table 5 and depicted in Figures 10, 11, and 12, indicated that the overall model was statistically significant (*p* < 0.05). The lack‐of‐fit values were insignificant for all responses, confirming the suitability and reliability of the model. Among the tested parameters, column temperature and flow rate had a prominent influence on R1, whereas both flow rate and organic ratio significantly affected R2 and R3. Nonetheless, these variations did not cause any meaningful change in resolution or overall method performance.

**TABLE 5:** ANOVA results.

Source	Sum of squares	*df*	Mean square	*F*‐value	*p*	
Response 1: resolution between impurities 2 and 4						
**Model**	5.7	3	1.9	31.83	< 0.0001	Significant
B‐Column temperature	0.7225	1	0.7225	12.09	0.0034	
C‐Organic ratio in M.P.A	4.62	1	4.62	77.38	< 0.0001	
bc	0.36	1	0.36	6.03	0.0268	
**Residual**	0.8961	15	0.0597			
Lack of fit	0.3861	5	0.0772	1.51	0.2693	Not significant
Pure error	0.51	10	0.051			
**Cor total**	6.6	18				
Response 2: resolution between impurities 4 and 3						
**Model**	5.84	3	1.95	26	< 0.0001	Significant
B‐Column temperature	0.6806	1	0.6806	9.09	0.0087	
C‐Organic ratio in M.P.A	4.31	1	4.31	57.49	< 0.0001	
bc	0.8556	1	0.8556	11.42	0.0041	
**Residual**	1.12	15	0.0749			
Lack of fit	0.4584	5	0.0917	1.38	0.3106	Not significant
Pure error	0.665	10	0.0665			
**Cor total**	6.97	18				
Response 3: resolution between impurity 3 and rifapentine						
**Model**	2.56	3	0.8542	23.98	< 0.0001	Significant
B‐Column temperature	1.82	1	1.82	51.16	< 0.0001	
C‐Organic ratio in M.P.A	0.49	1	0.49	13.76	0.0021	
bc	0.25	1	0.25	7.02	0.0182	
**Residual**	0.5343	15	0.0356			
Lack of fit	0.0843	5	0.0169	0.3749	0.8548	Not significant
Pure error	0.45	10	0.045			
**Cor total**	3.1	18				

The DoE outcomes (Table 6) confirmed that the analytical method remained stable and robust within the defined operational ranges, establishing a well‐defined method operable design region (MODR). In summary, the results demonstrated that the developed LC method is robust and dependable, ensuring consistent analytical performance under typical laboratory conditions.

**TABLE 6:** DoE data.

Std	Run	Factor 1 A: Flow rate (mL/min)	Factor 2 B: Column temperature (°C)	Factor 3 C: Organic ratio (%)	Response 1 Impurities 2 and 4	Response 2 Impurities 4 and 3	Response 3 Impurity 3 and rifapentine
9	1	0.8	25	110	4.4	4	7.4
15	2	1.2	35	110	4.1	3.9	7.8
7	3	1.2	35	90	5.4	5.2	8.2
2	4	0.8	25	90	5.6	5.4	7.4
17	5	1	30	100	4.8	4.6	7.6
3	6	1.2	25	90	5.4	5.2	7.3
16	7	1.2	35	110	3.9	3.7	7.8
10	8	0.8	25	110	4.2	3.8	7.3
13	9	0.8	35	110	4.1	3.9	7.2
8	10	1.2	35	90	4.5	4.1	8.3
5	11	0.8	35	90	4.6	4.2	8.5
19	12	1	30	100	4.8	4.6	7.6
14	13	0.8	35	110	3.9	3.9	8.1
6	14	0.8	35	90	4.6	4.2	8.3
12	15	1.2	25	110	4.1	3.8	7.3
18	16	1	30	100	4.8	4.6	7.6
11	17	1.2	25	110	3.8	3.6	7.2
4	18	1.2	25	90	5.4	5.2	7.4
1	19	0.8	25	90	5.6	5.4	7.5

*Note:* Response 1: Resolution between Impurities 2 and 4. Response 2: Resolution between Impurities 4 and 3. Response 3: Resolution between Impurity 3 and rifapentine.

## 4 Conclusion

A highly sensitive, precise, accurate, and robust liquid chromatographic method equipped with a photodiode array (PDA) detector was successfully developed and validated for the quantitative estimation of rifapentine‐related impurities in the rifapentine drug product. During method development, several analytical challenges were encountered, including insufficient resolution between closely eluting impurities with similar polarity, poor peak shape and symmetry, compromised peak purity, and interference from unknown co‐eluting compounds at the retention times of known impurities. These issues were systematically addressed through a rational, science‐based optimization process involving extensive evaluation of mobile phase compositions, pH adjustments, and selection of appropriate stationary phase chemistries.

Forced degradation studies were performed under various stress conditions, including acidic, basic, oxidative, and thermal environments, to evaluate the stability of the rifapentine formulation. The drug product exhibited notable degradation under these conditions, confirming the method's capability to effectively separate and quantify degradation products, thereby establishing its stability‐indicating nature. The optimized method was validated as per the ICH Q2(R2) guidelines and met all acceptance criteria for key performance parameters such as specificity, accuracy, precision, linearity, limit of detection (LOD), limit of quantification (LOQ), and robustness. Additionally, robustness was further verified using an analytical quality by design (AQbD)‐based approach, which involved systematic identification of critical method parameters (CMPs) and detailed risk assessment to ensure consistent method performance. In conclusion, the developed LC method is simple, cost‐effective, and dependable, making it highly suitable for routine quality control testing and stability studies of rifapentine drug products in pharmaceutical manufacturing and research settings.

## Author Contributions

**Siva Prasad Korikana:** formal analysis; validation; methodology; data curation. **Sreenivasa Rao Battula:** conceptualization; investigation; project administration; supervision. **Naresh Konduru:** investigation; supervision; writing – original draft; writing – review and editing. **Aravind Kurnool:** writing – original draft; writing – review and editing. **Divya Kumar Vemuri:** writing – original draft; writing – review and editing. **Venkata Lakshmana Sagar Dantinapalli:** writing – original draft; writing – review and editing.

## Funding

The authors have nothing to report.

## Conflicts of Interest

The authors declare no conflicts of interest.

## Supporting information


**FIGURE S1:** Method optimization trials – 1 chromatogram.
**FIGURE S2:** Method optimization trials – 2 chromatogram.
**FIGURE S3:** Method optimization trials – 3 chromatogram.
**FIGURE S4:** Method optimization trials – 4 chromatogram.
**FIGURE S5:** Ishikawa or fishbone diagram.
**FIGURE S6:** Accuracy study overlay chromatogram.
**FIGURE S7:** LOQ chromatogram (0.03% concentration).

## Data Availability

The data that support the findings of this study are available from the corresponding author upon reasonable request.
