# Supplementary material for: Quality by Design‐Based Development of a Robust LC Method for Simultaneous Estimation of Process‐ and Degradation‐Related Impurities in Rifapentine Drug Product for the Treatment of Active and Latent Tuberculosis
Source: Biomed Chromatogr. 2026 Mar 22;40(5):e70430. doi: 10.1002/bmc.70430 (PMC13006746; doi:10.1002/bmc.70430)
Supplement: Supplementary file 1 — FIGURE S1: Method optimization trials – 1 chromatogram. FIGURE S2: Method optimization trials – 2 chromatogram. FIGURE S3: Method optimization trials – 3 chromatogram. FIGURE S4: Method optimization trials – 4 chromatogram. FIGURE S5: Ishikawa or fishbone diagram. FIGURE S6: Accuracy study overlay chromatogram. FIGURE S7: LOQ chromatogram (0.03% concentration). [file BMC-40-e70430-s001.docx]

**Supplementary Materials**

**Quality by Design-Based Development of a Robust LC Method for Simultaneous Estimation of Process- and Degradation-Related Impurities in Rifapentine Drug Product for the Treatment of Active and Latent Tuberculosis**

Siva Prasad Korikana^1^, Sreenivasa Rao Battula^1*^, Naresh Konduru^1^, Aravind Kurnool^2^, Divya Kumar Vemuri^3^ Venkata Lakshamana Sagar Dantinapalli^4^

^1^Department of Chemistry, GITAM School of Science, GITAM (Deemed to be University), Visakhapatnam, Andhra Pradesh – 530045, India.

^2^Department of Chemistry, Sridevi Women’s Engineering College, Science and Humanities, Hyderabad, Telangana – 500075. India.

^3^Department of Chemistry, GITAM School of Science, GITAM Deemed to be University, Hyderabad, Telangana – 500081, India.

^4^Department of Chemistry, Raffles University, Neemrana, Alwar, Rajasthan - 301705, India.

***Correspondence:**

**Prof. Sreenivasa Rao Battula**, Department of Chemistry, GITAM School of Science, GITAM (Deemed to be University), Visakhapatnam, Andhra Pradesh – 530045, India.

Email: [sbattula2@gitam.edu](mailto:sbattula2@gitam.edu)

**
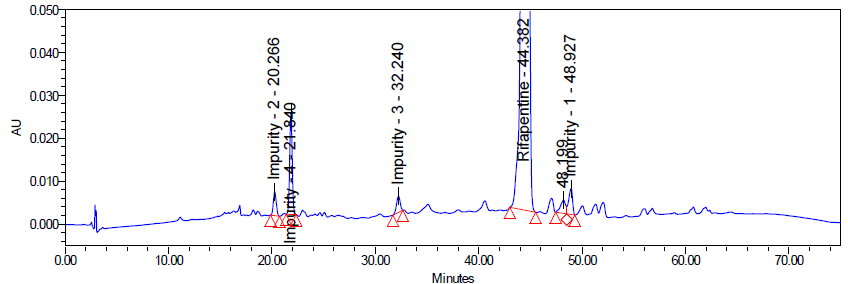
**

**Figure S1:** Method Optimization Trials – 1 Chromatogram

**
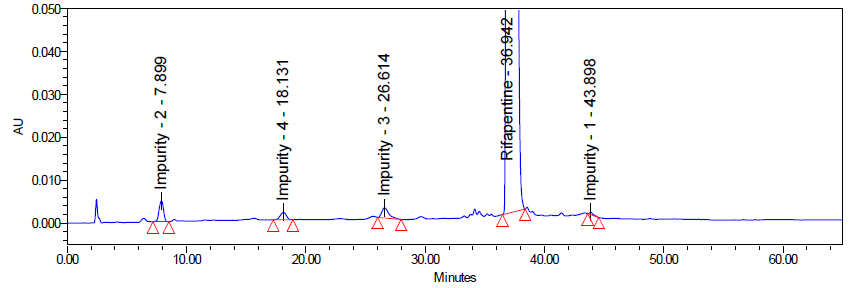
**

**Figure S2:** Method Optimization Trials – 2 Chromatogram

**
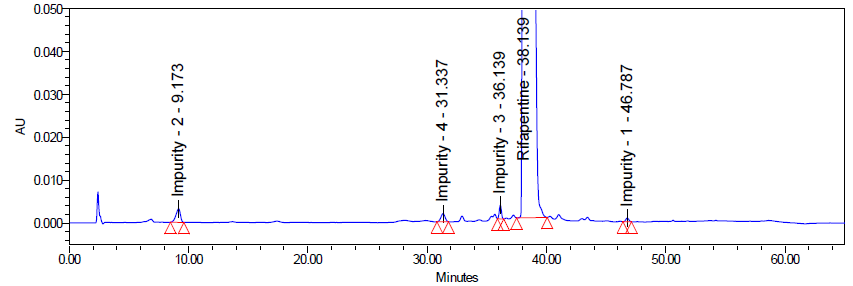
**

**Figure S3:** Method Optimization Trials – 3 Chromatogram

**
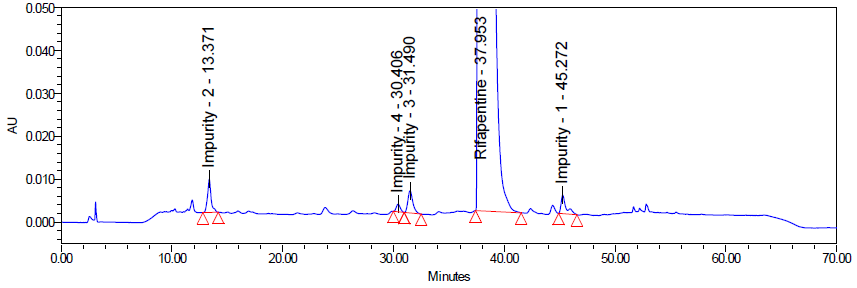
**

**Figure S4:** Method Optimization Trials – 4 Chromatogram


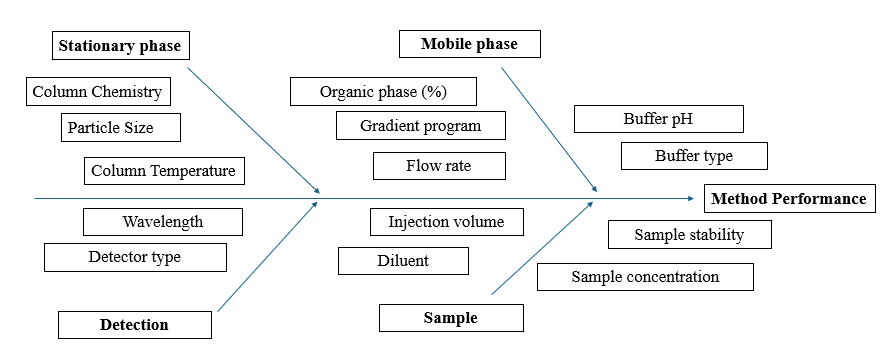


**Figure S5**: Ishikawa or fishbone diagram.


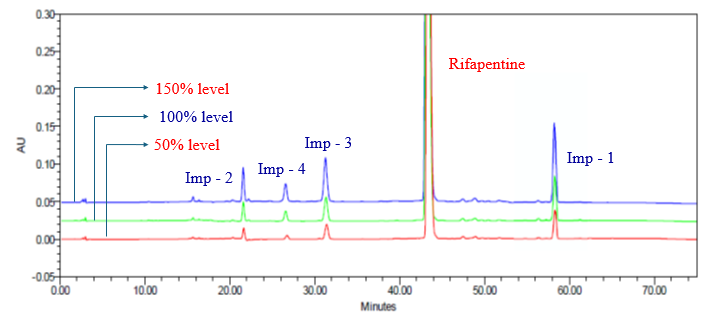


**Figure S6:** Accuracy study overlay chromatogram


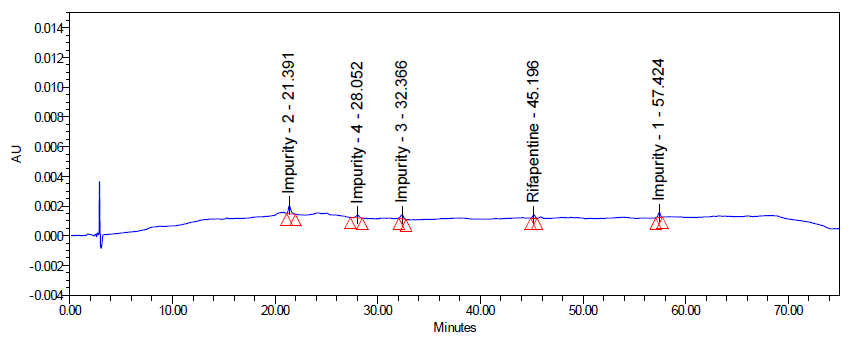


**Figure S7:** LOQ chromatogram (0.03% Concentration)
